# The Impact of Pesticide Use on Tree Health in Riparian Buffer Zone

**DOI:** 10.3390/toxics11030235

**Published:** 2023-02-28

**Authors:** K. Hanková, P. Maršík, T. Zunová, R. Podlipná

**Affiliations:** 1 Faculty of Agrobiology, Food and Natural Resources, Czech University of Life Sciences Prague, Kamýcká 129, Suchdol, 165 00 Praha 6, Czech Republic; 2 Laboratory of Plant Biotechnologies, Institute of Experimental Botany, Czech Academy of Sciences, Rozvojová 263, Lysolaje, 165 02 Praha 6, Czech Republic

**Keywords:** pesticides, chlorpyrifos, antioxidant defense system, poplar, hybrid aspen

## Abstract

The result of the enormous usage of pesticides in agriculture is the contamination of soil and water bodies surrounding the fields. Therefore, creating buffer zones to prevent water contamination is very useful. Chlorpyrifos (CPS) is the active substance of a number of insecticides widely used all over the world. In our study, we focused on the effect of CPS on plants forming riparian buffer zones: poplar (*Populus nigra* L., TPE18), hybrid aspen (*P.tremula* L. × *P. tremuloides* Michx.), and alder (*Alnus glutinosa* L.). Foliage spray and root irrigation experiments were conducted under laboratory conditions on in vitro cultivated plants. Spray applications of pure CPS were compared with its commercially available form—Oleoekol^®^. Although CPS is considered a nonsystemic insecticide, our results indicate that CPS is transferred not only upwards from roots to shoots but also downwards from leaves to roots. The amount of CPS in the roots was higher (4.9 times and 5.7 times, respectively) in aspen or poplar sprayed with Oleoekol than in those sprayed with pure CPS. Although the treated plants were not affected in growth parameters, they showed increased activity of antioxidant enzymes (approximately two times in the case of superoxide dismutase and ascorbate peroxidase) and augmented levels of phenolic substances (control plants −114.67 mg GAE/g dry tissue, plants treated with CPS—194.27 mg GAE/g dry tissue). In summary, chlorpyrifos, especially as a foliar spray pesticide, can create persistent residues and affects not only target plants but also plants surrounding the field.

## 1. Introduction

Although the use of pesticides in agriculture leads to increased crop yields, it also leads to an increased accumulation of these toxic substances in soil, water, and air [1]. The Eurostat (2022) published in its statistics about the sales of pesticides in the European Union (EU) that around 350,000 tons of pesticides are sold annually during recent years, mainly by four major producers (France, Germany, Italy, and Spain). Pesticides are often applied as sprays, so they move outside the application area with the wind. Protection of water bodies from this contamination is highly desirable. The most commonly used techniques include the creation of edge-of-field and riparian buffer zones, vegetated ditches, and artificial wetlands. Riparian buffer strips have already proved successful in reducing the nitrate load and removing pesticides [2]. When applied, pesticides undergo degradation via enzymatic reactions and rearrangements, microbial degradation, photolysis, abiotic hydrolysis, as well as inactivation due to binding to macromolecules or soil particles, which altogether leads to the generation of reactive oxygen species (ROS) in plant tissue [3].

Chlorpyrifos *O*,*O*-diethyl-*O*-(3,5,6-trichloro-2-pyridyl-phosphorothioate; CPS) is a crystalline, organophosphate insecticide [4]. It is widely used all around the world, and it is also the most commonly applied insecticide in the Czech Republic (approximately 182 t per year; [5])

Its major mechanism of action is the inactivation of acetylcholinesterase enzyme at the neural connections of insects. It is applied especially against widespread varieties of arthropods and harmful insects, e.g., mosquitoes (both larvae and hatches), soil and domestic pests, and ectoparasites on cattle, sheep, and poultry (ticks, lice, and bugs) [6]. It is also used to treat infested fruit trees and agricultural crops, especially sugar beet, fodder beet, corn, ornamental plants, cereals, peppers, kernels, cucumbers, tomatoes, potatoes, rapeseed, forests, and vines [7]. 

CPS is available in several forms that can be applied to foliage, such as wettable powders, emulsion concentrates, microcapsules, granules, or sprays. It can be applied before, during, or after crop planting, as well as during the dormant season [8]. Foliar-sprayed pesticides not only get caught on the leaves of the target plant, but they also hit the surrounding soil [9]. Next, volatilization from the foliage surface takes place, especially during the first 12 h, before the compound is adsorbed by foliage and soil [10]. The long-term persistence of CPS in the soil is caused by its high stability under neutral and acidic conditions [9]. CPS is toxic to nontarget aquatic organisms in the surrounding environment despite its relatively rapid translocation from the water to the sediment. The presence of its residues has been found in water as well as the sediments of many reservoirs or river basins [11]. It was reported that chlorpyrifos bioaccumulates in water organisms, such as blue-green algae, aquatic plants, goldfish, and mosquito fish [12]. Because of its potential genotoxicity and developmental neurotoxicity to humans, CPS is now banned from use on any crops sold in the U.S. and other 39 countries, including EU countries, Canada, Egypt, Indonesia, Turkey, etc. (The Consolidated List of Banned Pesticides; https://pan-international.org/pan-international-consolidated-list-of-banned-pesticides/, accessed on 16 January 2023). However, it is still used in many other countries, especially on the Asian continent, thus, its impact on the environment is still high. For example, recently published research in three locations of Kerman province in Iran demonstrated that residue levels of CPS in all Mazafati date fruits were above the maximum residue limits [13].

The uptake of CPS by plants was also investigated, and a reduction of growth was demonstrated [14,15,16]. Most often, the primary route by which pesticides are taken up by the plant and eventually translocated to the aboveground parts is their uptake by the roots from the aqueous solution in the soil pores [17]. The root uptake and translocation and accumulation of pesticides depend predominantly on pesticides’ polarity, dissociation constants, solubility, plant lipid content, and the pH of the surrounding soil and water [10]. In the study of Wang et al. (2016), *Acorus calamus* L. accumulated CPS from water bodies, and its content in plant tissues increased dose-dependently with the highest concentration of CPS in the roots [11]. Similarly, lettuce grown in soil treated regularly with CPS absorbed between 0.66 and 13.26 % of the initial concentration of CPS in the soil [9].

On the other hand, Ju 2020 et al. investigated the accumulation, translocation, and subcellular distribution of CPS in wheat and concluded that CPS was stored in roots without upward translocation. The amount of CPS in root cell walls (37.9–43.2%) and in organelles (43.1–47.6%) was significantly greater than its amount in the soluble fractions (13.2–16.1%). 

CPS also has a detrimental effect on plants. *Allium cepa* L. seedlings treated with CPS showed a dose-dependent inhibitory activity on several growth parameters. An augmentation of antioxidative enzyme activities may bypass the effect of pesticides to a certain extent, but it may vary in distinct plant species and for different pesticides [1]. CPS, at concentrations above 4 mg L^−1^, significantly inhibited the growth of *Iris pseudacorus* L. and caused a decrease in the efficiency of photosystem II and the photochemical quenching coefficient [18]. It also slightly inhibited the growth of water hyacinth (*Eichhornia crassipes* (Mart.) Solms) at concentrations between 0.5 and 1.0 mg L^−1^ [19]. 

In the experiments, we focused on the effect of CPS on plants forming riparian buffer zones, from which we chose poplar (*Populus nigra* L., TPE18), hybrid aspen (*P. tremula* L. × *P. tremuloides* Michx.), and alder (*Alnus glutinosa* L.). Poplar species are useful in a variety of environmental applications, for example, they can effectively remove, stabilize, and/or destroy contaminants in soil and groundwater due to their high growth rate, extensive root system, and high rate of soil water uptake [20]. An in vitro system simulating a contaminated stream was used. We studied the uptake of CPS and its effect on plants growing on a nutrient medium enriched with CPS. It is known that CPS in nature is transformed by microorganisms to 3,5, 6-trichloro-2-pyridinol (TCP), which has antimicrobial behavior, and then with the decrease of microbial community the speed of CPS degradation also declines. The in vitro system these circumstances overcomes and shows the clear metabolism by plants. The lack of information about the path of CPS inside the plant organism after foliage application led us to study the translocation of CPS from leaves to roots. With the aim of simulating more real situations in the field, we compared spray applications of pure CPS with its commercially available form Oleoekol® (Agrochemix, Rožňavská 17, 831 04 Bratislava, Slovakia) and also provided the experiment in a greenhouse. The experiments were provided inside the building in the cultivation room or a greenhouse under precisely controlled conditions in the area of IEB CAS, Prague. (50.12, 14.38).

## 2. Materials and Methods

### 2.1. Plant Material Cultivation and Experimental Design In Vitro

The in vitro cultures were chosen for these experiments because in vitro cultures offer a range of experimental advantages in studies aimed at examining the intrinsic metabolic capabilities of plant cells and their capacity for toxicity tolerance. The side effects of pesticides on the microbial community have long been known; we would like to study the ability to identify the contributions of plant cells to pollutant uptake and detoxification without interference from microorganisms [21,22]. Black poplar (*Populus nigra* L., TPE18) and hybrid aspen (*P. tremula* L. × *P. tremuloides* Michx.) regenerants obtained from the Forestry and Game Management Research Institute, Jíloviště, Czech Republic, were grown in vitro on hormone-free MS agar medium [23] (Table S1) at 25 °C with a 16 h photoperiod at 72 µmol of photons m^−2^ s^−1^ and were maintained by regular transplanting.

#### 2.1.1. Pilot Experiment with Poplar Cell Cultures

Cell cultures were obtained and cultivated as described previously [24]. Briefly, callus culture was obtained from hypocotyls of plants mentioned above by transplanting cuttings onto solid MS medium supplemented with the phytohormones 2,4-dichlorophenoxyacetic acid (0.225 mg L^−1^) and kinetin (0.215 mg L^−1^). The resulting callus cultures were grown in the dark at 25 °C. Suspension cultures derived from this callus were grown on the same medium as the callus cultures but not solidified with agar. Suspension cultures were grown in 250 mL flasks that were placed on a rotary shaker (120 rpm) To avoid exhaustion of some growth factors or accumulation of certain toxic metabolites in the culture medium, the suspension culture was subcultured every 2 weeks [25].

CPS (dissolved in dimethyl sulfoxide; DMSO) was added to the cultures so that the final concentration of CPS in the suspensions was 0.1 or 1 mg L^−1^. DMSO was also added to controls to achieve the same concentration of DMSO in all cultures. Cell suspensions were harvested on days 1, 4, and 8 after application in the exponential phase of suspension culture growth. The filtered cells were used to determine the activity of antioxidant enzymes and to analyze the CPS content. All samples were prepared in four biological replicates.

#### 2.1.2. Experiments with Plant Regenerants

In the first experiment, CPS was applied to the substrate. One week before the experiment, the 4-week old plantlets of hybrid aspen were transferred into liquid medium. Three doses of CPS were added in 7 days intervals at a concentration of 0.1 mg L^−1^ (predissolved in DMSO). The plants were harvested 7 days after the last application and divided into roots and shoots. Leaves (0.5 g) were immediately frozen in liquid nitrogen and consequently used for the determination of the activities of antioxidant enzymes, lipid peroxidation, and chlorophyll content. The roots and the rest of the leaves were dried by lyophilization and used for CPS content analysis.

In the second experiment, hybrid aspen and poplar plantlets were cultivated in vitro on medium without CPS, and CPS was applied on the leaf surface (spray experiment). The spraying was performed using an amber soda glass bottle with a pump vaporizer with a pump volume of 0.12 mL per stroke. We did not use only pure CPS but also the commercial insecticide Oleoekol where CPS is combined with a surfactant, 0.075% rapeseed oil methyl ester (RME). The plants were divided into four groups:

(1) Sprayed with DMSO in water (the same concentration as used for predissolution of the CPS);
(2) Sprayed with CPS (0.3 mg mL^−1^, predissolved in DMSO);
(3) Sprayed with 0.075% RME;
(4) Sprayed with Oleoekol at the manufacturer´s recommended concentration (corresponding to the concentration of the active substance (CPS) 0.3 mg mL^−1^ and surfactant (RME) 0.075%).

There were four Magenta boxes in each group. Every Magenta box contained one individual plant. One dose (0.250 mL) of appropriate solution was evenly sprayed into each box. All manipulation with the plants in boxes was performed aseptically in a laminar flow box (Aura HZ 48T, Bioair, S.p. A, Pero, Italy). The plants were harvested after 7 days, the roots were lyophilized, and the CPS content was measured. The leaves were used to determine the activities of antioxidant enzymes.

#### 2.1.3. Greenhouse Experiment

Alders (*Alnus glutinosa* L.) were grown in a greenhouse (23 °C, relative humidity about 60%). The plants were irradiated with sodium discharge lamps (400 W, ZG Lighting Czech Republic s.r.o.) with a 12 h photoperiod and an average irradiance of 72 μmol /m^2^/s on the surface of the plants, and horizontal differences in irradiance were <20%. Each plant was grown in separate pots on commercial compost Agro Profi from Agro CS a.s. (Říkov, Czech Republic). The plants were divided into four groups and sprayed with CPS (0.3 mg mL^−1^); 3) or Oleoekol as mentioned above.

### 2.2. Assays of Enzyme Activities

The plant cells (1 g) were ground to a fine powder and then suspended in (0.05 M) phosphate buffer composed of 0.1 mM EDTA; 1% PVP K 30; 0.5% Triton-X 100). After extraction (2 h), the homogenate was centrifuged at 10,000 g for 20 min and the supernatant was used for the determination of the protein content, which was determined according to [26], and the activities of the selected enzymes. Infinite M200 microplate reader (Tecan, Switzerland) was used for all spectrophotometric analyses. All the methods are well-known. The peroxidase activity (POD) was determined from kinetics of tetraguaiacol formation in a rection mixture containing 50 mM Tris HCl (pH 6), guaiacol (3.4 mM), and H_2_O_2_ (9 mM) [27]. The superoxide dismutase (SOD) activity was determined spectrophotometrically by measuring the ability of the enzyme to inhibit the photochemical reduction of nitro-blue tetrazolium (NBT) [28]. The enzyme activity was calculated by monitoring the reaction mixture for 120 s at 420 nm. The catalase (CAT) activity was assayed by measuring the initial rate of H_2_O_2_ disappearance using the method according to [29]; the peroxide decrease was displayed as a decline in absorbance at 240 nm. The ascorbate peroxidase (APX) activity was expressed as mmol of oxidized ascorbate as described by [30]. For the measurement of glutathion transferase (GST) activity, 1 mL reaction mixture contained 0.1 M sodium phosphate buffer (pH 6.5), 20 mL enzyme extract, and 2% 1-chloro-2,4-dinitrobenzene (CDNB). The enzyme activity was calculated by monitoring the reaction mixture at 340 nm as described by [31].

### 2.3. Chlorophyll Content

Fresh leaves were cleaned, and 0.25 g of crushed leaves was extracted in 10 mL methanol for 24 h. The content of photosynthetically active compounds (chlorophylls Chl a, Chl b, and carotenoids) of the filtered solution was measured using the classic spectrophotometric method with an Infinite M200 spectrophotometer (Tecan, Switzerland). Chlorophyll and carotenoid contents were calculated from the absorbance at 470, 652, and 665 nm according to [32].

### 2.4. Total Phenolic Content

Changes in the total phenolic content in the leaf extracts were chosen as stress markers and measured using the modified Folin–Ciocalteu method [33] as described previously [34,35]. Briefly, the extracts diluted in water in order to meet the calibration curve over LOQ were incubated with Folin–Ciocalteu reagent in 96-well microplates for 10 min while shaking (200 rpm) at room temperature. The reaction was terminated using 12% anhydrous sodium carbonate. The absorbance was read at 760 nm after 30 min incubation in the dark at 37 °C. The total phenol content was expressed in milligrams of gallic acid equivalent per gram of dry weight (mg GAeqv·g^−1^ DW) out of three individual measurements. The calibration curve was constructed with gallic acid in the concentration range from 1.172–25 µg mL^−1^ (R^2^ ≥ 0.99) with LOD = 4.82 ± 1.93 µg∙mL^−1^ and LOQ = 14.62 ± 5.86 µg∙mL^−1^.

### 2.5. LC/MS Analysis of Monitored Substances in the Plant

#### 2.5.1. Sample Preparation

The dried cells/leaves/roots were ground using liquid nitrogen. The samples (10 mg) were extracted with ethanol (15 mL), supplemented by butylated hydroxytoluene (BHT) and stable isotope (deuterium)-labeled chlorpyrifos (CPS-d10) as an internal standard for two hours with sonication. After centrifugation (4000× g/15 min/RT), the supernatant was separated and evaporated. Next, the samples were resuspended in 1 mL of 50% methanol and centrifuged again (14,000× g/ 20 min/RT). Before the measurement, the supernatant was filtered (Ø 0.22 µm).

#### 2.5.2. UHPLC/HRAM-MS Quantitation

The prepared samples were applied on an analytical system consisting of ultra-high performance liquid chromatography (UHPLC) Ultimate 3000 (Thermo-Fisher Scientific, Waltham, MA, USA) connected with a high-resolution accurate mass spectrometer (UHPLC/HRAM-MS, Orbitrap Exactive, Thermo-Fisher Scientific, Waltham, MA, USA). Methanol (100%) and water with 5 mM ammonium formate were used as mobile phases for gradient elution. The gradient began at 10% (0 min to 2 min) and then grew to 100% of A in 10 min, which was maintained until 15 min for washing of column, and finally was returned to initial conditions (10% A at 16 min) and kept for 4 min for system conditioning. The separation was performed using Kinetex Synergi Hydro-RP column (2.5 µm, 100 × 2.1 mm, Phenomenex, Torrance, CA, USA) tempered to 35°C and the flow rate set to 250 µL/min. The injection volume was 5 µL. The MS analysis was performed using positive ESI ionization and data was collected in FullMS/AIF scanning mode (resolution of 70,000 FWHM, m/z range from 170 to 900).

### 2.6. Statistical Analysis

To compare the changes in the treated and control plants, the data were processed using STATISTICA, CZ version 12.0 (StatSoft s.r.o., Prague, Czech Republic). One-way ANOVA followed by a post hoc Tukey’s test was used to prove the significant differences between the means of control and other treatments (*p* < 0.05).

## 3. Results and Discussion

### 3.1. Uptake of CPS by Poplar Cell Culture and Its Effect on Antioxidative Enzyme Activities

**Figure 1:**
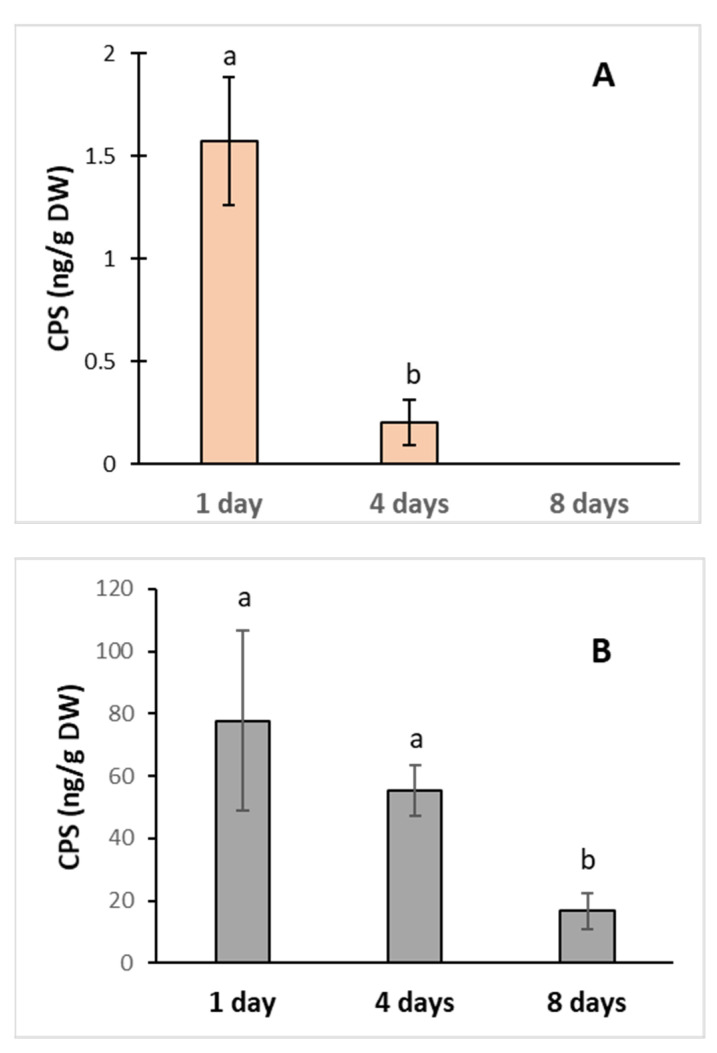
CPS content in poplar cells growing in medium supplemented with CPS at initial concentrations (**A**)—0.1 mg L^−1^; (**B**)—1 mg L^−1^. The samples were collected after 1, 4, and 8 days of cultivation. The data represent the mean ± S.D. (*n* = 4). Different letters mean statistically significant differences (*p* ≤ 0.05).

To study the effects of CPS on plants, we used the cell suspension of poplar (*Populus nigra*, TPE 18) as a model construct. Cells were grown in the medium supplemented with CPS in two concentrations, 0.1 and 1.0 mg L^−1^, for 8 days, resp. For both concentrations, the highest CPS levels were recorded 24 h after the treatment. Then, the levels of CPS in plants quickly decreased. In the case of an initial concentration of 0.1 mg, the amount of CPS after 8 days was under the detection limit (Figure 1). Both the viability of cells and the fresh mass were not affected by the tested CPS concentrations (data not shown). Rapid dissipation of CPS and found traces of the main product (TCP) proved the ability of poplar cells to uptake and metabolized CPS.

CPS toxicity was manifested by abnormal production of O^2−^ and H_2_O_2_ in CPS-treated *Brassica juncea* seedlings, which drastically increased O^2−^ by 70.5% and H_2_O_2_ by 45.7% compared to the control [16]. SOD starts the detoxification process by catalyzing the conversion of free radicals to molecular oxygen and hydrogen peroxide. H_2_O_2_ is then metabolized to water by ascorbate peroxidase (APX) and glutathione reductase (GR). This study revealed a significant stimulation of SOD activity of treated poplar cell suspensions after 8 days (for both CPS concentrations) (Table 1). Such stimulation may be a response to the accumulation of ROS, especially superoxide anions, after insecticide use. In accordance with these results, [36] observed a similar increase in SOD activity after the application of CPS to tomato plants and similarly [37] in their experiments with cucumbers. In our experiments, it was only SOD that was enhanced under CPS treatment. On the other hand, CAT activity, which is responsible for eliminating H_2_O_2_, is time-dependently and dose-dependently attenuated (Table 1). A decline in CAT activity is often observed under various stress conditions, while the activity of other antioxidant enzymes, such as SOD or GR, is usually induced by such stress treatments. It has been speculated that salicylic acid, which is formed during oxidative stress, causes a decrease in CAT activity in stressed plants [38]. CPS concentration (1 mg L^−1^) also caused a decrease in APX activity at all time points and POX activity on Days 1 and 4. Surprisingly, the least effect had the presence of CPS on the activity of GST, the enzyme of Phase II of plant metabolism of xenobiotics, which is responsible for the conjugation of several substrates, including pesticides, to form a nontoxic derivative (Table 1).

### 3.2. Uptake of CPS by In Vitro Cultivated Hybrid Aspen Regenerants and Its Effect on Pigment Content and Antioxidative Enzyme Activities

**Figure 2:**
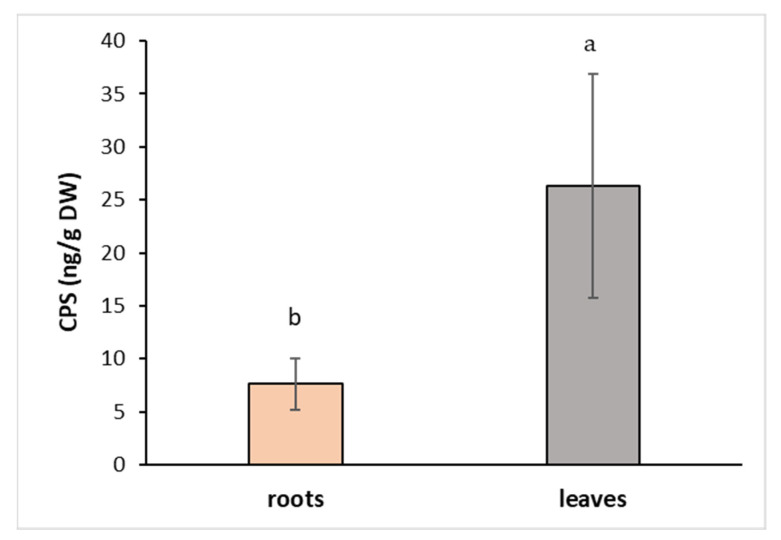
Content of CPS in hybrid aspen growing on medium supplemented with CPS at a concentration of 1 mg L^−1^. Samples were collected after 7 days of cultivation. Data represent the mean ± S.D. (*n* = 4). Different letters mean statistically significant differences (*p* ≤ 0.05).

The toxicity of CPS on different plant species has been first reported many years ago [39], and its effect on the reduction of growth has been tested, especially on crop plants and vegetables, to avoid yield reductions [10,36,40]. We have focused on the plants growing on the field margins to see how they cope with the presence of pesticides, including CPS, in their surroundings. A number of factors that are involved in the pesticide uptake and metabolism within the plant system include external environmental factors (temperature, humidity, pest presence, etc.) and physicochemical properties of the soil as well as pesticides. The CPS is known to affect the soil microbial biodiversity, which has a great impact on soil function and subsequently on plant growth [41]. To overcome the unpredictable constraints of nature, an in vitro system of cultivation was used. Hybrid aspen regenerants were cultivated in Magenta boxes on a nutrient medium supplemented with CPS at a concentration of 1 mg L^−1^ for 7 days. CPS was absorbed by the roots and then quickly transported to the aboveground parts. At the end of the experiment, the amount of CPS in the leaves was 3.5 times higher than its concentration in the roots (Figure 2). Lee et al. (2012) demonstrated that poplar and willow trees have a strong ability to uptake CPS and translocate it within plants. In contrast with our results, they observed a higher accumulation of CPS in the roots than in the leaves. This could be due to the higher initial concentration (25 mg L^−1^) that they used [42]. 

**Figure 3:**
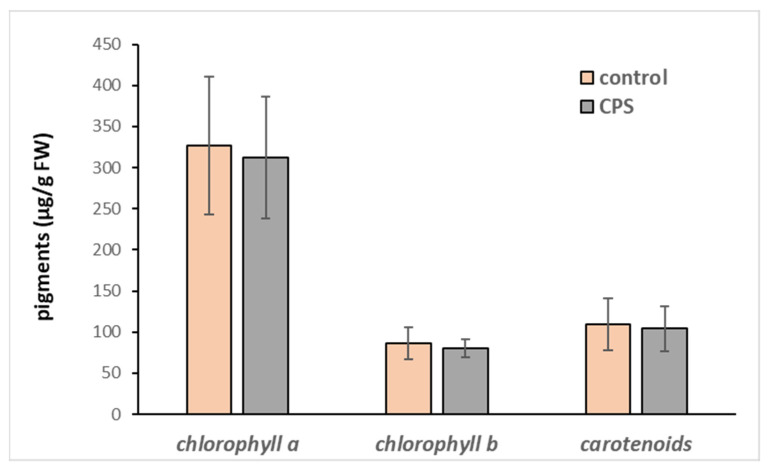
Chlorophyll *a*, chlorophyll *b,* and carotenoid content in hybrid aspen growing in medium supplemented with CPS at an initial concentration of 1 mg L^−1^. Samples were collected after 7 days of cultivation. The data represent the mean ± S.D. (*n* = 4). The statistical analysis did not show any significant difference (*p* < 0.05).

**Figure 4:**
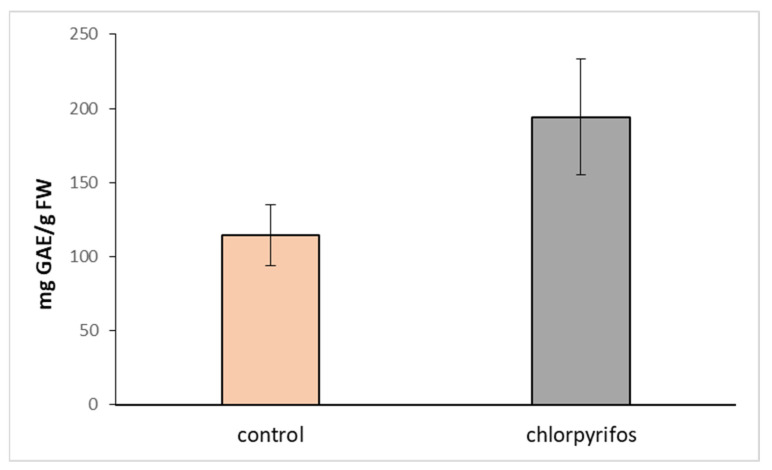
Total phenol content—TPC (mg GAE/g dry tissue) in the leaves of hybrid aspen. Comparison of the content in plants growing for 7 days in the medium supplemented with chlorpyrifos (CPS) at an initial concentration of 1 mg L^−1^ and control plants. Data represent the mean ± S.D. (*n* = 4). The statistical analysis showed a significant difference (*p* < 0.05).

Upon treatment with CPS, we did not observe any morphological changes in plants, such as necrosis, chlorosis, stunting, burns, or twisting of leaves. Therefore, the possible toxicity of this pesticide was determined based on the changes in the activity of the antioxidant enzymes and in the content of total phenolic compounds and photosynthetic pigments. While the content of chlorophyll *a*, *b,* and carotenoids have not been affected (Figure 3) and the activities of most measured antioxidant enzymes only slightly declined (Table 2), the amount of total phenolic compounds increased significantly (Table 2, Figure 4). 

Total phenol content, the nonenzymatic part of the plant defense system, was reported to be augmented under abiotic stress similar to the presence of pesticides, salts, or heavy metals [43]. The reactions of antioxidant enzymes to pesticide treatment are influenced by the tested plant species, the duration of the treatment, the applied concentration of the pesticide, etc. A decrease in the activities of antioxidant enzymes, especially at high concentrations of pesticides, might be associated with accelerated O^2−^ production and the enhancement of membrane lipid peroxidation as described by [44].

### 3.3. CPS Application on Plant Leaves

In the first experiment, poplars and hybrid aspens were cultivated in vitro, and CPS was applied to the leaves by spraying. Four groups were used as described in Materials and Methods. Although nonsystemic pesticides such as CPS tend to be lipophilic and absorbed into the waxy cuticle of plants, our results showed that both plant species translocated CPS from the leaves to the roots. This process could be partly a consequence of incomplete cuticle development in in vitro plants.

**Figure 5:**
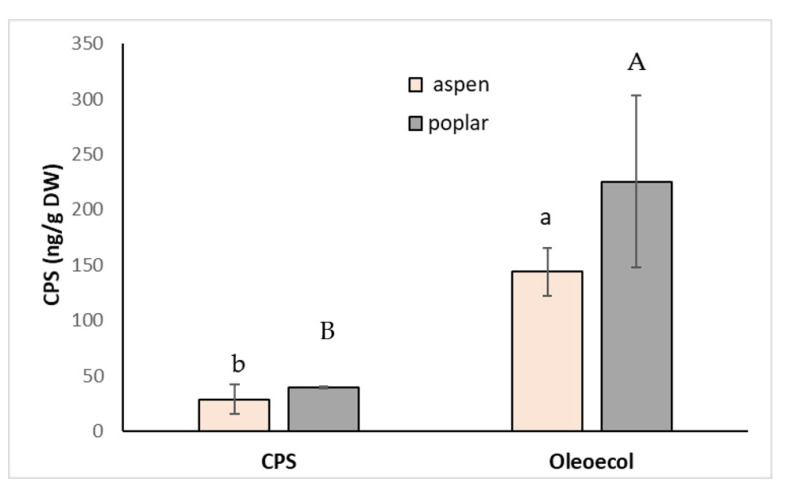
Concentration of CPS in the roots of poplar and hybrid aspen sprayed with 0.250 mL solution of CPS (3 mg L^−1^) or the insecticide Oleoecol (0.250 mL) at the producer´s recommended concentration. Samples were collected 10 days after foliage application. Different letters mean statistically significant differences (*p* ≤ 0.05).

For example, an unexpectedly high level of CPS in apple pulp was explained by the existence of microfissures in the skin due to pest or abiotic damage [45]. The amount of CPS in the roots was higher (4.9 times and 5.7 times, respectively) in aspen and poplar sprayed with Oleoekol, probably due to better absorption because of the presence of emulsifier RME (Figure 5). The differences between plant species were not statistically significant. Even in this experiment, the pesticide application did not significantly affect plant morphological parameters. 

**Figure 6:**
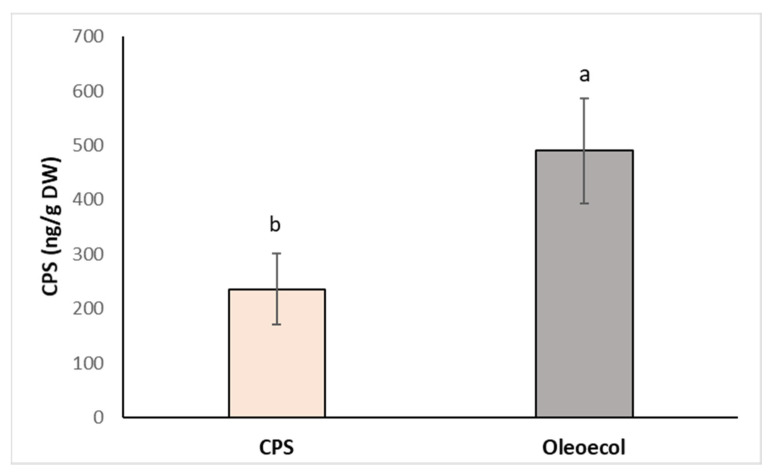
Concentration of CPS in the roots of alder sprayed with 0.750 mL solution of CPS (3 mg L^−1^) or the insecticide Oleoecol (0.750 mL) at the producer’s recommended concentration. Samples were collected 10 days after foliage application. Different letters mean statistically significant differences (*p* ≤ 0.05).

In the second case, a foliar spray of CPS or Oleoecol was applied to alder grown in the greenhouse. Three doses (0.750 mL) of CPS or Oleoecol solution were applied to the surface of the seedling leaves. Ten days after application, the plants were harvested and the chlorpyrifos content in the roots was determined. Although the CPS concentration in the Oleoecol dose was also 3 mg L^−1^, the CPS concentration in the roots after spraying with Oleoecol was more than twofold higher compared to spraying with pure CPS solution (Figure 6). This experiment confirmed the results obtained in the in vitro system, that the plants are able to move CPS from aboveground part to roots. Nowadays, published study [37] also described the translocation of CPS from leaves to roots in cucumber via phloem.

The shifts in the activities of the antioxidant enzymes in the poplar plants 10 days after foliage application of CPS or Oleoekol were followed in order to determine the toxicity effect. In contrast to the application of CPS into the substrate, the activities of all antioxidant enzymes increased (Table 3). These results are in accordance with [40], who described an increase in the activities of antioxidant enzymes after surface spraying of CPS or other pesticides on the foliage of spinach, as well as with the results obtained by [46] who observed a general increase in the activities of all measured antioxidant enzymes in wheat grown on promethrin-treated soil. Moreover, mung bean seedlings treated with CPS showed a significant enhancement of SOD activity [47]. Because peroxidases are preventing/prevent the accumulation of H_2_O_2_, it is not surprising that the activity of APX and POX also increased. Additionally, [48] described that rimsulfuron caused significant increases in APX activity in broad bean and maize species.

The presented results indicate that RME itself may be a significant stressor. We found the enhancement of APX, SOD, and POX activities in the group of plants sprayed only with RME (Table 3). Similarly, the negative impact of concentrated RME on barley growth was described in the study of [49]. 

## 4. Conclusions

In the last few decades, the use of pesticides in the agricultural industry has become almost unavoidable. Therefore, it is important to minimize the impact on nontarget organisms and the environment, for example, by creating buffer zones around water bodies. The selection of appropriate plant species is essential for the proper functioning of such a zone. Our results showed the ability of poplars and aspens to take up and accumulate the insecticide chlorpyrifos in relatively high concentrations without visible damage. However, when compared to the control plants, treated plants showed increased activity of antioxidant enzymes as well as increased content of phenolic compounds, which can be considered a sign of stress. It is also important to mention the translocation of CPS occurs not only from roots to shoots when it is applied to substrate medium but also from leaves to roots when applied using foliage spray. Furthermore, the amount of CPS in the roots was five times higher in plants sprayed with commercial product Oleoekol than with pure CPS, which highlights the significant role of RME in CPS absorption.

## Figures and Tables

**Table 1:** Activity of antioxidant enzymes (APX—ascorbate peroxidase; SOD—superoxide dismutase; CAT-catalase; POX—peroxidase; GST—glutathione-S-transferase) in poplar cells after 1, 4, and 8 days of exposure to CPS concentrations of 0.1 and 1 mg L^−1^. Data are expressed as the percentage of controls (=100%) representing the mean ± S.D. (*n* = 4). Values that show statistically significant (*p* < 0.05) deviations from the control are in bold.

0.1 mg CPS	APX			SOD			CAT			POX			GSH		
control	100.0	±	*10.6*	100.0	±	*4.7*	100.0	±	*3.2*	100.0	±	*8.1*	100.0	±	*22.3*
1.day	99.5	±	*3.9*	**51.8**	±	*8.1*	**66.7**	±	*17.9*	**57.0**	±	*22.8*	107.7	±	*1.1*
4.day	102.2	±	10.1	88.1	±	20.6	**47.8**	±	1.3	78.1	±	21.3	**62.0**	±	3.1
8.day	**56.5**	±	15.2	**236.5**	±	8.2	**27.6**	±	2.4	**82.9**	±	2.5	99.9	±	20.3
**1 mg CPS**	**APX**			**SOD**			**CAT**			**POX**			**GSH**		
control	100.0	±	*10.6*	100.0	±	*4.7*	100.0	±	*3.2*	100.0	±	*8.1*	100.0	±	*22.3*
1.day	**57.2**	±	*3.8*	90.8	±	*9.0*	**45.8**	±	*8.1*	**54.1**	±	*7.1*	88.0	±	*1.0*
4.day	**62.0**	±	15.6	**69.8**	±	7.8	**19.0**	±	4.3	**54.1**	±	7.8	80.2	±	1.4
8.day	**19.8**	±	8.0	203.6	±	13.5	**17.2**	±	5.8	89.8	±	4.8	86.6	±	5.3

**Table 2:** Activity of antioxidant enzymes (APX—ascorbate peroxidase; SOD—superoxide dismutase; CAT—catalase; POX—peroxidase; GST—glutathione-S-transferase) in leaves of the hybrid aspen after 7 days of exposure to 1 mg L^−1^ CPS. The data are expressed as the percentage of controls (=100%) representing the mean ± S.D. (*n* = 4). Values that show statistically significant (*p* < 0.05) deviations from the control are in bold.

	APX			SOD			CAT			POX			GST		
control	100.0	±	18.4	100.0	±	38.3	100.0	±	19.5	100.0	±	35.6	100.0	±	38.5
CPS	72.0	±	14.0	51.3	±	11.9	**66.8**	±	11.4	54.0	±	13.8	81.3	±	13.6

**Table 3:** Activity of antioxidant enzymes (APX—ascorbate peroxidase; SOD—superoxide dismutase; CAT—catalase; POX—peroxidase; GST—glutathione-S-transferase) in leaves of the poplar 10 days after spraying with CPS (3 mg L^−1^) in water solution, by rapeseed oil methyl ester (RME) solution or Oleoecol including CPS (3 mg L^−1^), and RME. The data are expressed as the percentage of controls (=100%) representing the mean ± S.D. (*n* = 4). Values that show statistically significant (*p* < 0.05) deviations from the control are in bold.

	APX			SOD			CAT			POX			GST		
control	100.0	±	30.5	100.0	±	17.0	100.0	±	4.5	100.0	±	48.2	100.0	±	19.8
CPS	**139.7**	±	8.1	**158.3**	±	27.8	**209.0**	±	68.9	129.4	±	48.7	**154.2**	±	9.7
RME	**164.5**	±	2.2	**157.2**	±	37.8	102.2	±	12.3	**217.2**	±	53.2	98.3	±	28.7
Oleoecol	**216.8**	±	37.9	**173.9**	±	63.1	106.2	±	7.7	172.3	±	54.2	81.9	±	9.3

## Data Availability

The data presented in this study are available on request from the corresponding author.

## Supplementary Materials

The following supporting information can be downloaded at: https://www.mdpi.com/article/10.3390/toxics11030235/s1, Table S1: Composition of *Murashige and Skoog* Basal *Medium*.
