# Supplementary material for: The Impact of Pesticide Use on Tree Health in Riparian Buffer Zone
Source: Toxics. 2023 Feb 28;11(3):235. doi: 10.3390/toxics11030235 (PMC10053419; doi:10.3390/toxics11030235)
Supplement: Supplementary file 1 [file toxics-11-00235-s001.zip › toxics-2197081-supplementary.pdf]

toxics

MDPI

Supplementary Materials: The Impact of Pesticide Use on Tree Health in Riparian Buffer Zone

Table S1. Composition of *Murashige and Skoog* Basal *Medium*.

**Macronutrients**	
Ammonium nitrate (NH_4NO_3)	1650 mg/L
Calcium chloride ($CaCl_2 \cdot 2H_2O$)	440 mg/L
Magnesium sulfate ($MgSO_4 \cdot 7H_2O$)	370 mg/L
Monopotassium phosphate (KH_2PO_4)	170 mg/L
Potassium nitrate (KNO_3)	1900 mg/L
**Micronutrients**	
Boric acid (H_3BO_3)	6. 2 mg/L
Cobalt chloride ($CoCl_2 \cdot 6H_2O$)	0.025 mg/L
Ferrous sulfate ($FeSO_4 \cdot 7H_2O$)	27.8 mg/L
Manganese(II) sulfate ($MnSO_4 \cdot 4H_2O$)	22.3 mg/L
Potassium iodide (KI)	0.83 mg/L
Sodium molybdate ($Na_2MoO_4 \cdot 2H_2O$)	0.25 mg/L
Zinc sulfate ($ZnSO_4 \cdot 7H_2O$)	8.6 mg/L
FeNaEDTA	36.70 mg/L
Copper sulfate ($CuSO_4 \cdot 5H_2O$)	0.025 mg/L
**Vitamins and organic compounds**	
Myo-Inositol	100 mg/L
Nicotinic Acid	0.5 mg/L
Pyridoxine · HCl	0.5 mg/L
Thiamine · HCl	0.1 mg/L
Glycine	2 mg/L
saccharose	30g/ L
